# Aortic arch variant: Dual anomalous origins of the left vertebral and left internal mammary artery

**DOI:** 10.1016/j.xjse.2025.100066

**Published:** 2025-08-05

**Authors:** Gopidi Aparanji, Nagaraja Moorthy, Anand Palakshachar, Rajendra Prasad, Manidipa Majumdar

**Affiliations:** a Department of Cardiology, Sri Jayadeva Institute of Cardiovascular Sciences and Research, Bangalore, Karnataka, India

**Figure:**
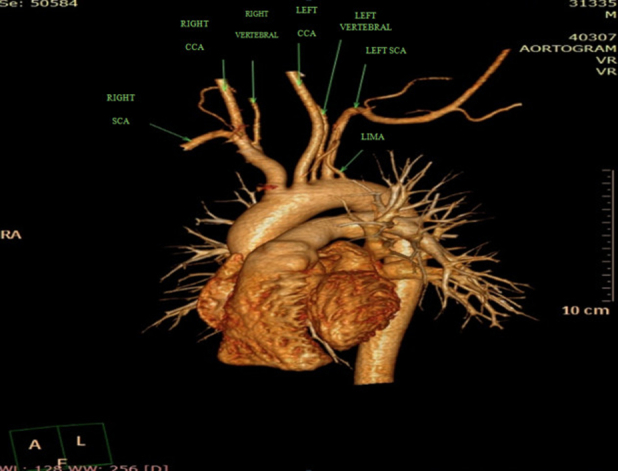
CTA showing the separate origin of left vertebral and LIMA from the aortic arch.

**Central Message**

A unique case of coronary artery disease with LIMA and vertebral artery arising directly from arch of the aorta—unusual aortic arch anatomical variant.

A 48-year-old man with diabetes and hypertension underwent coronary angiography for acute coronary syndrome. Findings on angiogram revealed distal left main stenosis and triple-vessel disease, and the patient was planned for coronary artery bypass graft surgery (CABG). As per institutional protocol, left internal mammary artery (LIMA) imaging was performed preoperatively. Left subclavian angiography did not reveal LIMA at its expected origin (Figure 1, *A*, Video 1). However, faint retrograde opacification suggested a possible anomalous origin from the aorta. Selective engagement revealed a normally coursing LIMA arising directly from the aorta (Figure 1, *B*, Video 2), along with a large side branch most probably intercostal branch. Computed tomography aortography further confirmed the anomalous origin of LIMA from the aortic arch distal to the left subclavian artery as seen in the reconstructed intraluminal image (Figure 2, Video 3). Another interesting variation was even left vertebral artery was arising directly from aorta medial to left subclavian artery. He underwent successful CABG and on regular follow-up.

**Figure 1:**
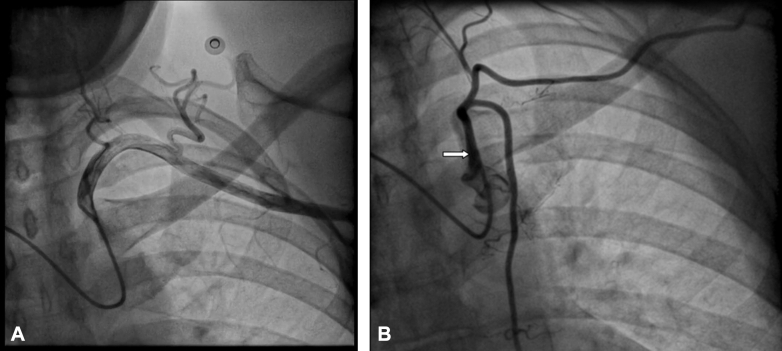
A, LIMA could not be identified with conventional angiogram of left subclavian artery. B, Selectively engaged LIMA (*white arrow*) arising directly from the aorta. *LIMA*, Left internal mammary artery.

**Figure 2:**
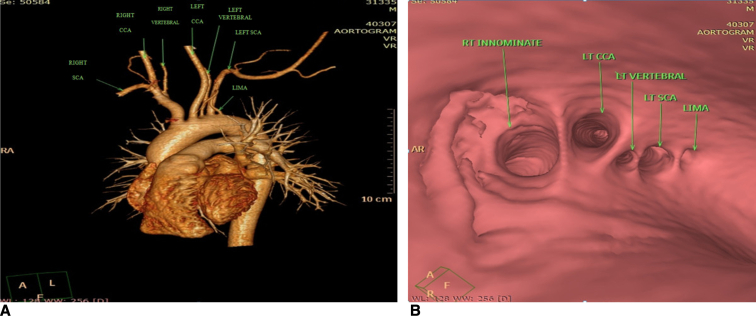
A, Computed tomography angiography (CTA) showing the separate origin of the left vertebral and left internal mammary artery directly from aortic arch. B, 3-dimensional reconstructed image of aortic arch branches.

Typically, LIMA arises from the proximal left subclavian artery in 92% of cases, from the midportion in 7%, and the distal segment in 1%. Rare origins include the junction of the left subclavian and aorta,^1^ an aberrant vertebral artery,^2^ or a thyrocervical trunk arising from the arch.^3^ Anomalous vertebral artery origin is also uncommon, occurring in 2% to 6% of individuals. Early identification of anomalous vessels is critical to avoid intraoperative challenges with graft access and thoracic aortic pathology. In previous reports, anomalous LIMA origins were often recognized only during post-CABG angiography and vertebral artery variants during cadaver studies. In our case, anomalous origin of LIMA was detected during coronary angiography, and it was engaged selectively before arch aortography. To best of our knowledge, this is the first case of dual anomalous origin of LIMA and vertebral artery from the aortic arch diagnosed before CABG surgery in the literature. This case illustrates the potential variable origin of the LIMA and highlights the critical importance of performing non selective aortic arch angiography before concluding that a LIMA graft is occluded as routine angiographic assessment of LIMA is not performed prior to CABG in many centers. Informed consent was obtained from the patient for the publication of this case report; institutional review board approval was not required. The supporting data including images and video files relating to the case can be made available by the primary and corresponding authors on request.

## Conflict of Interest Statement

The authors reported no conflicts of interest.

The *Journal* policy requires editors and reviewers to disclose conflicts of interest and to decline handling or reviewing manuscripts for which they may have a conflict of interest. The editors and reviewers of this article have no conflicts of interest.

## Supplementary Data

Video 1 Conventional angiogram of left subclavian artery using 6-F Judkins right catheter in anteroposterior view did not show left internal mammary artery at its expected origin. Video available at: https://www.jtcvs.org/article/S2950-6050(25)00025-7/fulltext.

Video 2 Selective intubation of left internal mammary artery arising directly from the aortic arch distal to the left subclavian artery with normal course of the rest of the artery. Video available at: https://www.jtcvs.org/article/S2950-6050(25)00025-7/fulltext.

Video 3 Three-dimensional reconstruction of computed tomography angiography showing five aortic arch branches, in sequence: right innominate artery, left common carotid artery, left vertebral artery arising directly from the aortic arch medial to the left subclavian artery, left subclavian artery, and left internal mammary artery arising directly from the aortic arch distal to the left subclavian artery. Video available at: https://www.jtcvs.org/article/S2950-6050(25)00025-7/fulltext.
