# Supplementary figures and images for: Aortic arch variant: Dual anomalous origins of the left vertebral and left internal mammary artery
Source: JTCVS Struct Endovasc. 2025 Aug 5;7:100066. doi: 10.1016/j.xjse.2025.100066 (PMC13244770; doi:10.1016/j.xjse.2025.100066)

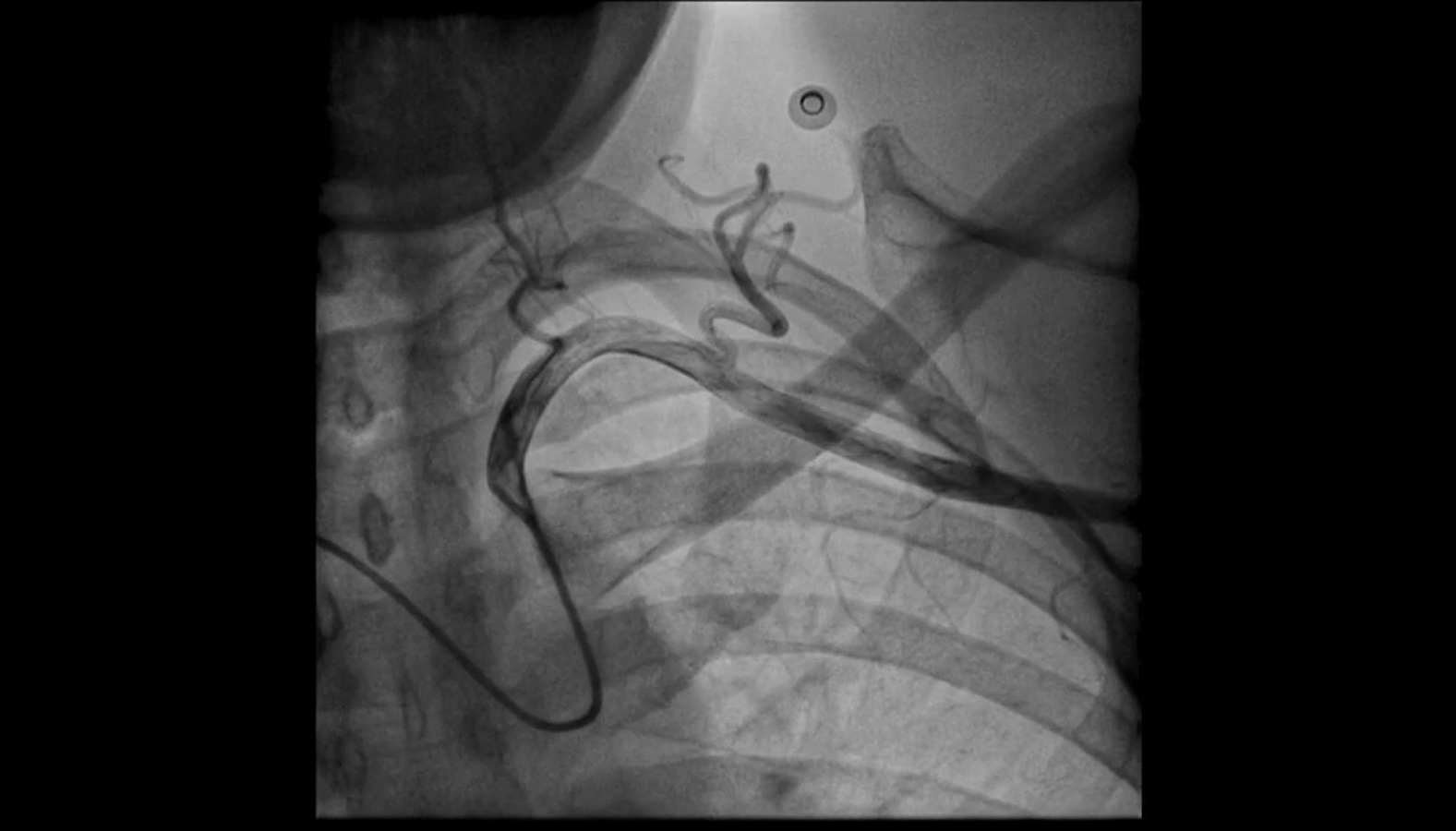

Supplement: Video 1 — Conventional angiogram of left subclavian artery using 6-F Judkins right catheter in anteroposterior view did not show left internal mammary artery at its expected origin. Video available at: https://www.jtcvs.org/article/S2950-6050(25)00025-7/fulltext. [file fx2.jpg]

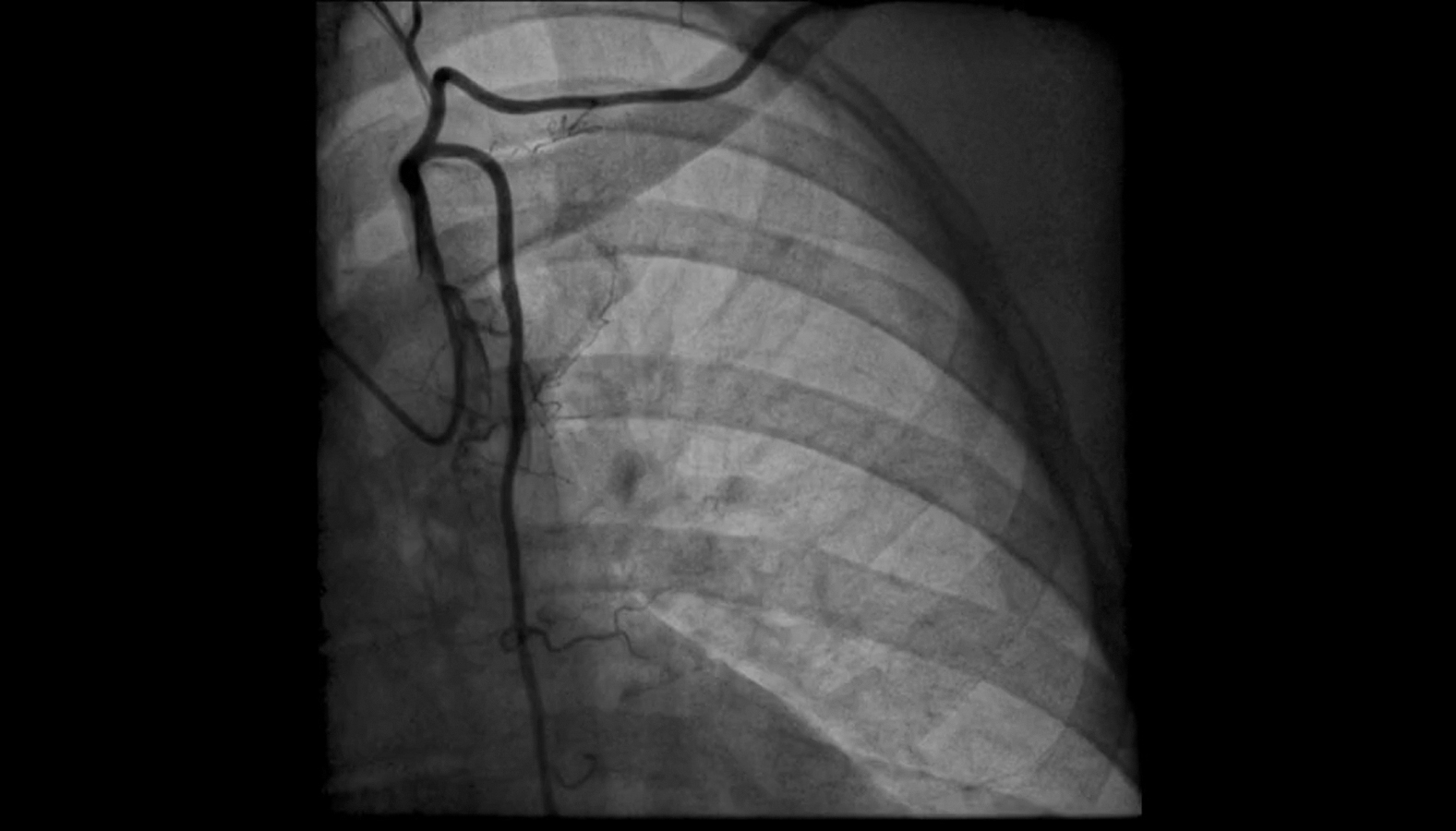

Supplement: Video 2 — Selective intubation of left internal mammary artery arising directly from the aortic arch distal to the left subclavian artery with normal course of the rest of the artery. Video available at: https://www.jtcvs.org/article/S2950-6050(25)00025-7/fulltext. [file fx3.jpg]

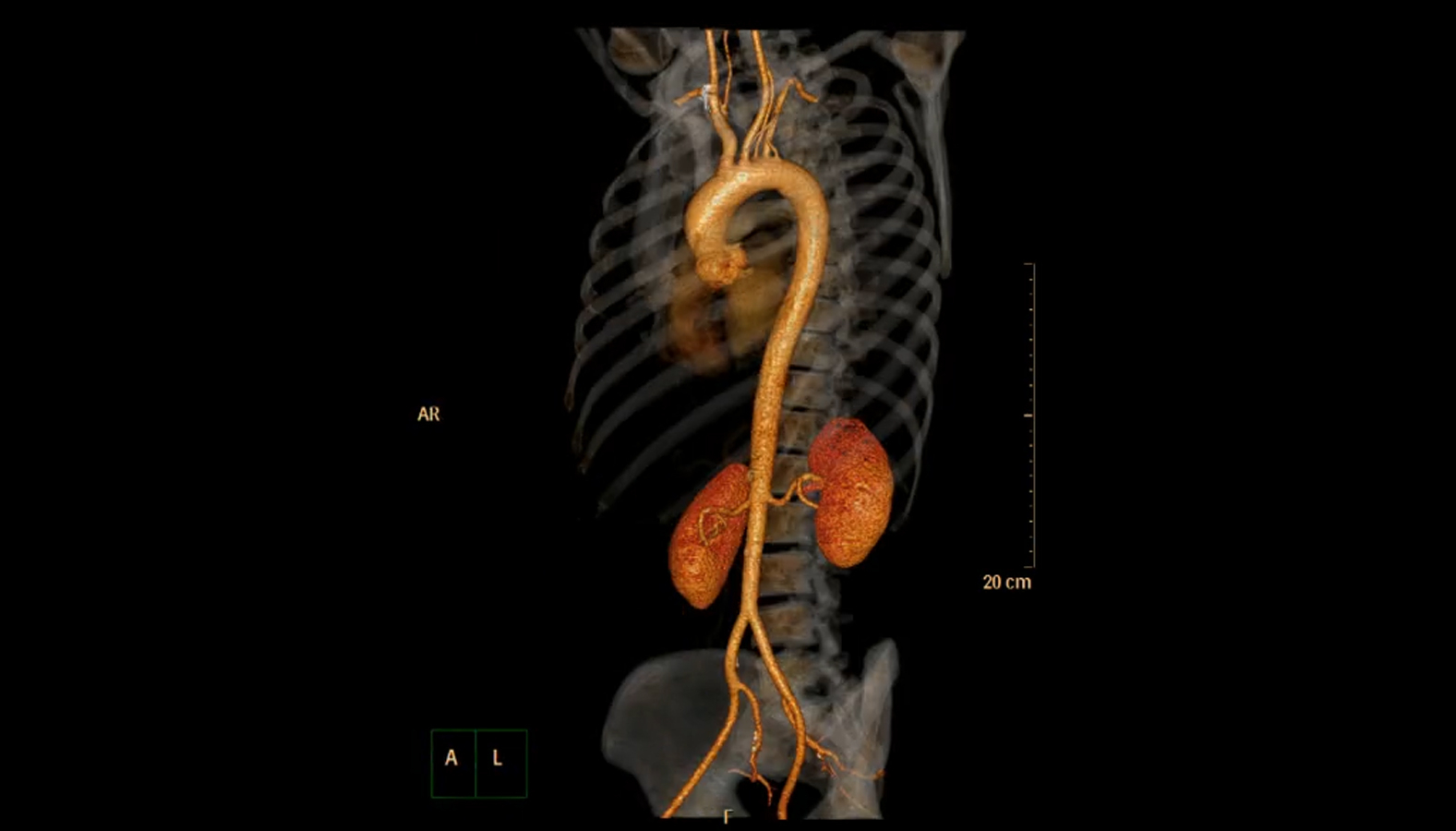

Supplement: Video 3 — Three-dimensional reconstruction of computed tomography angiography showing five aortic arch branches, in sequence: right innominate artery, left common carotid artery, left vertebral artery arising directly from the aortic arch medial to the left subclavian artery, left subclavian artery, and left internal mammary artery arising directly from the aortic arch distal to the left subclavian artery. Video available at: https://www.jtcvs.org/article/S2950-6050(25)00025-7/fulltext. [file fx4.jpg]
